# Pharmacokinetics of fluralaner as a systemic drug to control infestations of the common bed bug, *Cimex lectularius*, in poultry facilities

**DOI:** 10.1186/s13071-023-05962-3

**Published:** 2023-09-21

**Authors:** Maria A. González-Morales, Andrea E. Thomson, James Yeatts, Hiroko Enomoto, Ahmed Haija, Richard G. Santangelo, Olivia A. Petritz, Rocio Crespo, Coby Schal, Ronald Baynes

**Affiliations:** 1 Department of Entomology and Plant Pathology, North Carolina State University, Raleigh, NC USA; 2 Department of Clinical Sciences, College of Veterinary Medicine, North Carolina State University, Raleigh, NC USA; 3 Department of Population Health and Pathobiology, College of Veterinary Medicine, North Carolina State University, Raleigh, NC USA

**Keywords:** *Cimex*, Bed bugs, Fluralaner, Pharmacokinetics, Poultry, Ectoparasites

## Abstract

**Background:**

Bed bug infestations are re-emerging in the poultry industry throughout the USA. Although the impacts of bed bugs on birds’ health and welfare are poorly understood, adverse outcomes are expected, including stress, anemia, infections and lower production rates. Worker welfare is also an important consideration in commercial poultry farms. A limited number of insecticides are available for use in the complex spatial environment of commercial farms. Systemic drugs have the potential to overcome the limitations of existing pest management tactics. A recent study showed that fluralaner administered to chickens caused high levels of mortality in bed bugs.

**Methods:**

To further understand the efficacy of this approach, we evaluated the pharmacokinetics of an oral solid formulation of fluralaner in 11 chickens and quantified its plasma concentration in chickens using UPLC/MS. We administered fluralaner to chickens with two doses of Bravecto^®^ (each 0.5 mg/kg body mass) via gavage 1 week apart and evaluated its efficacy on bed bugs that fed on medicated chickens for up to 28 days post-treatment.

**Results:**

Bed bugs that fed on fluralaner-treated chickens experienced > 50% mortality within 30 min of the administration of Bravecto and 100% mortality 2 days post-treatment. Mortality slowly declined to 66.6% by day 28. Fluralaner was quantifiable in the hens’ plasma for at least 28 days post-treatment. The treatment resulted in maximal plasma concentrations (*C*_max_) of 106.4 ng/ml around day 9.0 (*T*_max_), substantially higher than the LC_90_, the concentration needed to kill 90% of the bed bugs.

**Conclusions:**

Fluralaner appears to be a promising candidate for bed bug control in poultry farms, with a treatment effect lasting at least 28 days.

**Graphical Abstract:**

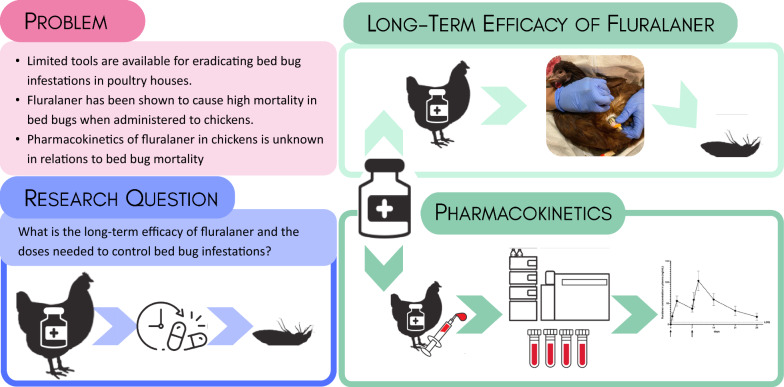

## Background

Bed bugs (*Cimex lectularius*) are ectoparasitic obligate hematophagous insects that feed exclusively on warm-blooded hosts such as humans, bats and birds [1]. They were first reported in the poultry industry in the USA in the 1940s [2] and as a major poultry pest in 1985 [3]. Bed bugs have limited dispersal capabilities, and their introduction into residential settings is usually human-mediated and involves small, genetically inbred propagules [4]. Likewise, introductions of bed bugs into poultry farms are facilitated by humans [3]. The impacts of bed bug infestations on chicken health and welfare are understudied; however, it is likely that bed bug bites, like those of other ectoparasites, cause skin reactions and infections, itchiness, feather pecking and overall lower production due to stress and possibly anemia [5, 6].

After being nearly eradicated using DDT, organophosphates and carbamates in the mid-to-late 1900s [7], in the past few decades bed bug infestations have resurged in the poultry industry [7, 8]. Few insecticides in few formulations are labeled to control bed bugs in poultry farms and, along with insecticide resistance and structural constraints, these factors impede efforts to eradicate infestations in poultry farms. Nevertheless, the recent use of veterinary drugs to control bed bugs in laboratory settings has shown promising results [9, 10]. For example, fluralaner, a member of the isoxazoline class of insecticides (IRAC Class 30), caused high mortality, including in multi-resistant bed bug strains [10]. Insecticidal and acaricidal activity of isoxazolines is due to a dual mode of action as inhibitors of the gamma-aminobutyric acid (GABA)-gated chloride (GABACl) channels and glutamate-gated chloride (GluCl) channels and their greater affinity for invertebrate receptors than mammalian receptors [11]. First introduced in 2014, fluralaner is a common ectoparasitic drug used in dogs and cats. Although currently there is no fluralaner formulation available for use in poultry in the USA, in Europe Exzolt^™^ is approved for mite control [12]. Fluralaner is the only isoxazoline approved for poultry, and it represents a new group of active ingredients for bed bug control with promising potential of controlling pyrethroid- and neonicotinoid-resistant bed bugs.

In light of the limited availability of formulations for use in poultry farms, we administered Bravecto to hens and monitored its efficacy on bed bugs [10]. Although this formulation performed well, based on the in vitro laboratory-estimated LC_50_ value and the time-course of bed bug mortality on medicated chickens, we suspected that the pharmacokinetics of fluralaner in the Bravecto formulation might differ appreciably from the pharmacokinetics reported for Exzolt in hens [12]. Therefore, this study aims to (i) test the efficacy of ingested fluralaner (Bravecto) in egg laying chickens and (ii) evaluate the pharmacokinetics of fluralaner in chicken plasma to understand the effectiveness of the active ingredient over time.

## Methods

### Experimental insects and rearing procedures

The Harlan strain of *C. lectularius* was collected at Fort Dix, New Jersey (USA), in 1973 and maintained on a human host until 2008. Between 2008 and 2021, it was maintained in our laboratory on defibrinated rabbit blood and since 2021 on human blood. The Harlan strain is a reference insecticide-susceptible colony that has not been exposed to pesticides since its collection. Bed bugs were maintained at 25 °C, 50 ± 5% relative humidity and a photoperiod of 12:12 (light:dark) h, and reared in plastic jars (118-cm^3^) containing Manila cardstock paper substrate for harborage and capped with plankton netting (BioQuip Products, Rancho Dominguez, CA, USA) to enable aeration and feeding. Bed bugs were fed weekly on heparinized human blood supplied by the American Red Cross (IRB #00000288 and protocol #2018-026) delivered through an artificial feeding system. The artificial feeding system, as shown in [10] and modified after [13] and [14], was housed in a North Carolina State University (NCSU)-approved BSL-2 facility (Biological Use Authorization # 2020-09-836). Plant grafting tape (A.M. Leonard Horticultural Tool and Supply Co., Piqua, OH, USA) was stretched across the bottom of the feeder and served to hold the blood within each feeder, functioning as a membrane through which bed bugs could feed.

### Birds

A flock of 11 Rhode Island Red hens (*Gallus gallus domesticus*) (range of body weight: 2.0–3.3 kg; mean weight: 2.5 kg), ranging in age from 1 to 2 years, was used in this study. Birds were housed as a group in a climate-controlled facility (15.6 m^2^) with wooden shavings substrate and maintained on a 12:12 (light:dark) h cycle. The flock was obtained from a private supplier and maintained on a Layer NCSU diet with water available ad libitum through automatic waterers. Bird health was monitored daily, and optimum welfare conditions were based on serial physical examinations, fecal floatation, serial packed cell volumes via microhematocrit tube and centrifugation, serial total solids via refractometer and serial biochemical panels (VetScan Avian/Reptile Profile Plus, Abaxis Inc., Union City, CA, USA). All procedures were authorized by the NCSU Institutional Animal Care and Use Committee (IACUC # 21-152).

### Efficacy of fluralaner on bed bugs when administered to chickens

All 11 birds were treated with fluralaner, but randomly split into two groups. Six birds were used for pharmacokinetic analysis (blood drawn) and also exposed to bed bugs to assess bed bug mortality over time. The remaining five birds were used exclusively for pharmacokinetic analysis, and these birds were not fed upon by bed bugs.

There is no commercial product containing fluralaner labeled for poultry use in the USA. Therefore, we used the oral formulation of fluralaner, Bravecto, registered for miniature dogs, and adjusted the dose by weighing portions of Bravecto chewable tablets, based on the mass of each hen. Portions of Bravecto were administered via gavage at a dose of 0.5 mg/kg body mass based on a protocol approved by regulatory authorities in Australia and the European Union for use of a fluralaner-containing product on chickens (Exzolt, MSD Animal Health, Germany) [12]. Fluralaner was administered at baseline [day 0; treatment 1 (T1)] and again 7 days later [day 7; treatment 2 (T2)].

Chickens were assessed nine times for either bed bug mortality or pharmacokinetics, or both. Except for the blood draws and subsequent pharmacokinetic analysis, we followed precisely the procedures outlined in [10], so direct statistical comparisons could be made with the previous treatment group. The time course was as follows: (i) before treatment control (30 min before fluralaner administration, Pre-T1); (ii) 30 min after the fluralaner administration (0.5 h Post-T1); (iii) 2 days after treatment (2 days Post-T1); (iv) 7 days after treatment (7 days Post-T1); (v) 30 min after the administration of the second dose on day 7 (7 days Post-T1 = 0.5 h Post-T2); (vi) 9 days after treatment (9 days Post-T1 = 2 days Post-T2); (vii) 14 days after treatment (14 days Post-T1); (viii) 21 days after treatment (21 days Post-T1); (ix) 28 days after treatment (28 days Post-T1).

Birds were held individually on our lap and gently restrained with a towel to minimize stress. Harlan strain adult male bed bugs (15 insects per replicate), 7 days after feeding on human blood, were contained in clear 20-ml plastic containers. The plankton netting of the bed bug container was gently placed on the lateral inguinal region of each chicken. Bed bug groups were allowed to feed for 10 min. Each fully engorged bed bug was placed individually in a well of a 24-well cell culture plate and maintained in an incubator at the same rearing conditions described above. Each chicken was exposed to a single bed bug group (maximum 15 bed bugs) on each given day, and subsequent feedings were alternated between the left and right inguinal region to avoid any potential discomfort or bias. Bed bug mortality was assessed every 24 h for up to 7 days post blood-feeding on a treated bird by gently touching individual insects with entomological forceps, categorizing them as alive (coordinated avoidance movement) or dead (no response or unable to right themselves after touching with forceps).

### Pharmacokinetics of fluralaner

Eleven birds were treated with 0.5 mg/kg body mass of fluralaner (Bravecto) at baseline (day 0) and again on day 7, and they were evaluated by collecting blood samples at the time points indicated above. Five of these birds were not exposed to bed bugs, and in the six chickens that were evaluated in bioassays with bed bugs, blood was collected immediately after each bed bug feeding session. Blood was collected using a 26-gauge needle and 1-ml syringe, alternating collections from a leg (medial metatarsal), wing (ulnar veins) and jugular of each bird. Birds were manually restrained during blood collection. Blood samples were centrifuged (3500 ×*g* for 6 min) within 1 h after collection and stored at − 20 °C until assayed.

#### Solvents and reagents

Technical grade fluralaner was purchased from Cayman Chemical (Ann Arbor, MI, USA) and used as a standard in developing and validating the extraction and UPLC/MS procedures. Acetonitrile Optima LC–MS grade, methanol Optima LC–MS grade, formic acid Optima LC–MS grade, and o-phosphoric acid (85wt%) HPLC grade, were purchased from Fisher Scientific (Thermo Fisher, Waltham, MA, USA). Ultrapure (type 1) water was obtained from a Millipore Synergy UV water purification system (Millipore Sigma, Burlington, MA, USA).

#### Sample preparation

Two hundred microliters of the plasma was diluted with 500 µl 4% phosphoric acid in water and then loaded onto a Waters Oasis PRIME HLB 1 cc (30 mg) solid-phase extraction (SPE) cartridge (Waters Corp., Milford, MA, USA). The SPE cartridge was then washed with 1 ml 95:5 water:methanol. The fluralaner was eluted with 1 ml of 90:10 acetonitrile:methanol, evaporated to dryness at 55 °C and then reconstituted in 300 µl 50:50 methanol:water. The sample was vortex mixed briefly, filtered with Whatman Mini-UniPrep^™^ syringeless filter devices containing PVDF filter media (Cytiva, Marlborough, MA, USA) and then analyzed by ultra performance liquid chromatography coupled to mass spectrometry (UPLC/MS).

#### UPLC/MS conditions

Analysis was performed on a Waters Acquity Ultra Performance Liquid Chromatograph coupled to a Waters Acquity QDa mass spectrometer detector (Waters Corp., Milford, MA, USA). The instrument was set to Single Ion Recording (SIR) of 554.0473 m/z using electrospray ionization in the negative ion mode (ESI-). The cone and capillary voltages were 25 V and 0.8 V, respectively. A Waters Acquity UPLC BEH Phenyl 1.7 µm (2.1 mm × 100 mm) column with corresponding VanGuard Pre-Column (2.1 mm × 5 mm) was used for the separations. A gradient was used for the mobile phase. Solvent A was 0.1% formic acid in water. Solvent B was 0.1% formic acid in acetonitrile. The flow rate was 0.40 ml/min. The gradient was programmed as follows: from 0 to 0.50 min the mobile phase composition was 60% A and 40% B; from 0.50 to 2.50 min the composition changed linearly to 10% A and 90% B then held there until 3.50 min; finally, back to 60% A and 40% B at 3.51 min and held to 5.00 min.

The fluralaner concentration range was 5 to 1000 ng/ml with a correlation coefficient, *R*^2^, of 0.99. The limit of detection (LOD) was 2 ng/ml. The limit of quantification (LOQ) was 5 ng/ml. Intra- and interday precision and accuracy are shown in Table 1a and b, respectively. Intraday precision and accuracy were obtained by using four different concentrations repeated five times each on the same day. Interday precision and accuracy were obtained by measuring six different concentrations on seven different days.

**Table 1:** Intraday precision and accuracy (a) and (b) interday precision and accuracy

a. Intraday precision and accuracy				
Spiked concentration (ng/ml)	Average fluralaner concentration (*n* = 5)	Standard deviation (ng/ml)	Relative standard deviation (RSD %)	Average accuracy (%)
5	5	0.4	7.4	98.2
10	10	0.3	2.6	103.6
50	44	2.9	6.5	88.7
1000	1088	47.4	4.4	108.8

b. Interday precision and accuracy				
Spiked concentration (ng/ml)	Average fluralaner concentration (*n* = 7)	Standard deviation (ng/ml)	Relative standard deviation (RSD %)	Average accuracy (%)
5	5	0.5	9.8	101.0
10	9	0.3	3.6	94.3
50	49	1.2	2.5	98.5
100	99	5.8	5.8	99.2
500	524	39.3	7.5	104.8
1000	1008	60.8	6.0	100.8

### Data analysis

Differences in mortality of bed bugs that fed on the fluralaner-treated birds were determined using linear mixed model with repeated measures [based on restricted maximum likelihood (REML)] and Tukey’s HSD test [15]. Means are presented with standard error of the mean. A non-compartmental analysis of fluralaner in chicken plasma was conducted (Phoenix WinNonlin, version 8.3, Certara, St. Louis, MO, USA). The pharmacokinetic parameters were estimated for fluralaner in plasma after gavage administration and included the elimination rate constant (λz), terminal half-life (HL_λz), time to peak concentration (*T*_max_), peak concentration (*C*_max_), area under the curve from time zero to the last time point (AUC_last_), area under the curve from time zero to the infinity (AUC_inf_), extrapolation of AUC (AUC_extrap_), volume of distribution per fraction absorbed (Vz_F), clearance per fraction absorbed (Cl_F) and the mean residence time (MRT). These values were calculated using the linear log trapezoidal method. The LOQ values (5 ng/ml) were included for the pharmacokinetic analysis. Two birds (one from each group) were excluded because of high AUC_extrap_ (> 30%), which did not pass the criteria of non-compartmental analysis. Differences between the two groups of chickens (*n* = 4 and 5 birds) were analyzed using Student’s t-test (2-tailed).

## Results

### Bed bug feeding assays on fluralaner-treated chickens

Cumulative percent mortality on day 7 was used to evaluate efficacy because treated bed bugs showed high mortality, while control bed bugs that had fully fed on untreated chickens showed low mortality [10]. After each feeding, we also collected blood samples from the Bravecto-treated chickens for pharmacokinetic analysis.

**Fig. 1:**
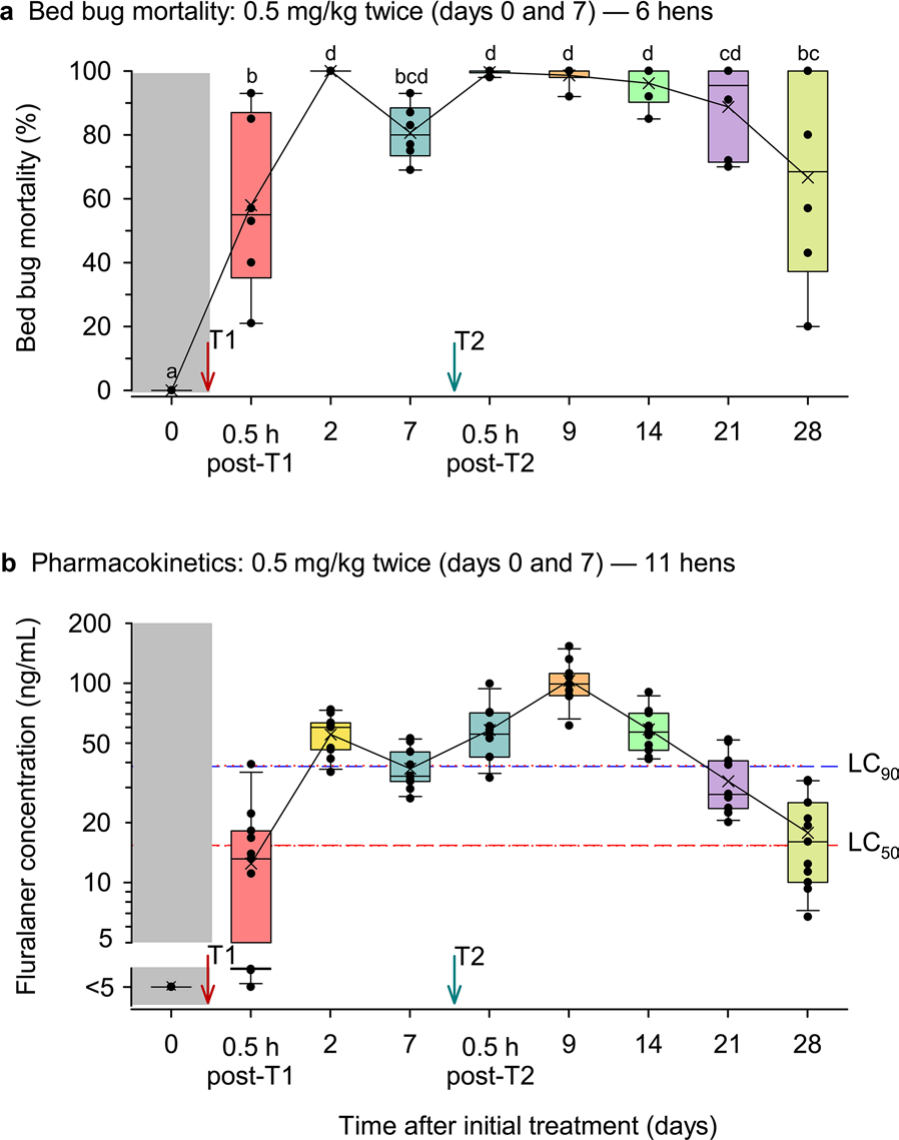
Mortality of bed bugs that fed on chickens treated with fluralaner and the concentration of fluralaner in chicken blood during 28 days after treatment. **a** Bed bug mortality. Six chickens were treated with 0.5 mg/kg body mass on day 0 and again on day 7 (T2, Treatment 2). Up to 15 bed bugs were fed on each bird at each time point, with each time point represented by 78–87 bed bugs (out of a maximum of 90 bed bugs) that fed to repletion. A linear mixed model (based on restricted maximum likelihood) was conducted within each experiment followed by Tukey’s honestly significant difference test to separate means (represented within box plots by X). Means with different lowercase letters (above box plots) are significantly different at *α* = 0.05. **b** Fluralaner concentration in chicken blood (note log-scale). Blood from 11 chickens was collected from the 6 chickens used in **a** and 5 chickens that were not exposed to bed bugs. The LOQ was 5 ng/ml. The LC_50_ (15.3 ng/ml) and LC_90_ (38.6 ng/ml) lines were derived from dose–response curves of fluralaner-supplemented blood fed to bed bugs in artificial feeders [10]

Bed bug mortality was significantly higher at all time points after Bravecto treatment (0.5 h to 28 days) than before the gavage administration on day 0 (linear mixed model, *F*_5,8_ = 25.3550, *P* < 0.0001; Tukey’s HSD) (Fig. 1a). The first peak in bed bug mortality (100%) was 2 days after the first treatment and mortality remained high through 21 days after this treatment (14 days post T-2). By day 28, however, mean mortality significantly declined to 66.6 ± 9.2% (*P* < 0.05).

The design of this experiment, with six chickens treated twice with 0.5 mg fluralaner/kg body mass, was identical to our previously reported experiment with six chickens (Fig. 5b in the 2022 study [10]), but we used a younger flock in the present experiment. We also drew blood from these chickens for pharmacokinetic analysis. Overall, the two experiments yielded similar results. However, in the present experiment bed bug mortality lagged somewhat, at 55.8 ± 11.3% 0.5 h after the first fluralaner treatment, compared to 72.5 ± 13.7% in the previous study [10]. Likewise, bed bug mortality declined to approximately 65% by day 28 in both experiments, but the variation across the six replicates was greater in the present experiment than in [10].

### Pharmacokinetics of fluralaner in chickens

All 11 birds were treated with fluralaner and then randomly divided into two groups. Six birds were used for pharmacokinetic analysis, where blood samples were taken, and they were also exposed to bed bugs to observe bed bug mortality over time. The remaining five birds were used solely for pharmacokinetic analysis and were not fed upon by bed bugs. We calculated all parameters separately and compared the groups to confirm there was no statistically significant difference that could be attributed to exposure to bed bugs (Table 2). At 0.5 mg/kg body mass administered to chickens twice, fluralaner was quickly absorbed into the blood, and it continued to be well above the LOQ in plasma for at least 28 days (Fig. 1b, Table 2). We found no significant differences between the two groups of five and four chickens, so the data were combined (*n* = 9). The peak concentration was 2 days after the first treatment, and the overall peak concentration (*C*_max_) was 106.4 ± 1.2 ng/ml at *T*_max_ of 9.0 days (2 days after the second treatment) (Table 2). The mean concentration of fluralaner declined gradually to 17.79 ng/ml 28 days after the initial treatment. The AUC was 988 ng*day/ml (days 0 to 28) and 1159 ng*day/ml (days 0 to infinity), suggesting that extrapolation to infinity was 12.5%, which is acceptable. Other pharmacokinetic parameters are shown in Table 2.

**Table 2:** Comparison of plasma pharmacokinetic parameters after gavage administration of fluralaner (0.5 mg/kg body mass on day 0 and again on day 7) in 9 chickens

pK parameters	Unit	Group 1: Geometric mean ± standard deviation (coefficient of variation) *n* = 5	Group 2: Geometric mean ± standard deviation (coefficient of variation) *n* = 4	*P*-value^k^	Combined Geometric mean ± standard deviation (coefficient of variation) *n* = 9
R^2^		0.99 ± 1.0 (0.93)	0.99 ± 1.0 (1.1)	0.935	0.99 ± 1.0 (0.9)
λz^a^	1/day	0.09 ± 1.4 (34.0)	0.11 ± 1.2 (17.4)	0.207	0.098 ± 1.3 (30.1)
HL_λz^b^	Day	8.0 ± 1.4 (34.0)	6.1 ± 1.2 (17.4)	0.142	7.1 ± 1.3 (30.1)
T_max_^c^	Day	9.0 ± 0.0 (0.0)	9.0 ± 0.0 (0.0)	1.000	9.0 ± 0.0 (0.0)
C_max_^d^	ng/ml	105.2 ± 1.2 (15.2)	107.8 ± 1.3 (26.3)	0.805	106.4 ± 1.2 (19.3)
AUC_last_^e^	ng*day/ml	1071.1 ± 1.2 (20.5)	893.3 ± 1.2 (17.6)	0.194	988.1 ± 1.2 (20.5)
AUC_inf_^f^	ng*day/ml	1313.3 ± 1.3 (29.0)	990.6 ± 1.2 (15.4)	0.111	1158.6 ± 1.3 (27.2)
AUC_extrap_^g^	%	15.7 ± 1.9 (74.8)	9.4 ± 1.4 (33.5)	0.106	12.5 ± 1.8 (62.9)
Vz_F^h^	l/kg	4.4 ± 1.2 (18.7)	4.4 ± 1.3 (28.7)	0.892	4.4 ± 1.2 (21.9)
Cl_F^i^	l/kg/day	0.4 ± 1.3 (29.1)	0.5 ± 1.2 (15.3)	0.103	0.4 ± 1.3 (27.3)
MRT^j^	Day	12.0 ± 1.4 (31.4)	9.1 ± 1.1 (11.9)	0.102	10.6 ± 1.3 (27.7)

^a^*λz* elimination rate constant

^*b*^*HL_λz* terminal half life

^c^*T*_*max*_ time to the peak concentration

^d^*C*_*max*_ peak concentration

^e^*AUC*_*last*_ area under the curve from time zero to the last time point

^f^*AUC*_*inf*_ area under the curve from time zero to infinity

^g^*AUC*_*extrap*_ extrapolation of AUC

^*h*^*Cl_F* clearance per fraction absorbed

^i^*Vz_F* volume of distribution per fraction absorbed

^j^*MRT* mean residence time

^k^No statistically significant differences were found in any parameter between the two groups of chickens (two-tailed t-tests, *α* = 0.05)

## Discussion

The dependency of bed bugs on host-feeding makes systemic veterinary antiparasitic drugs particularly appropriate to consider for bed bug control. The use of veterinary drugs on companion and farm animals has been highly effective for controlling various pests, including fleas, ticks, mosquitoes and mites. Bed bugs have re-emerged in poultry farms throughout the USA, and infestations represent a serious and intensifying problem in the poultry industry [7, 10]. A limited number of active ingredients is labeled for bed bug control in this challenging environment, and insecticide resistance is further limiting the suitability of some insecticides.

In a recent study, we supplemented blood in an artificial feeder with technical fluralaner and demonstrated that the LC_50_ and LC_90_ values in fully engorged bed bugs were 15.3 and 38.6 ng/ml, respectively [10]. Several field-collected bed bug strains with high resistance to pyrethroid insecticides were likewise susceptible, with little evidence of cross-resistance to fluralaner. We then treated two independent flocks of chickens with Bravecto and demonstrated high bed bug mortality for at least 28 days post-treatment [10]. However, the plasma concentrations of fluralaner were not measured, so we could not relate the LC values to plasma concentrations of fluralaner. Therefore, in this study we repeated the treatment regime that we used before, but also quantified plasma concentrations of fluralaner in chickens treated with Bravecto. Thus, this is the first study to explore the pharmacokinetics of fluralaner as a potential systemic drug to control bed bugs as ectoparasites of chickens.

The pattern of bed bug mortality that we observed was similar to what we observed in the previous study [10]. Mortality of bed bugs increased to > 50% within 30 min of the first administration of Bravecto to chickens (0.5 h post T1, Fig. 1a), and 100% of the bed bugs that fed on chickens 2 days post-treatment died. Following the second Bravecto administration on day 7 (T2), > 90% of the bed bugs died through day 14, and subsequently the effects of fluralaner slowly declined.

A pharmacokinetic study following oral administration of Exzolt to hens reported > threefold higher plasma concentrations of racemic fluralaner than in our study, with a *C*_max_ of 355.1 ng/ml at 7.5 days [12]. Based on the mortality results in our previous study [10], we predicted that treatments with Bravecto might result in lower blood concentrations than with Exzolt and thus substantially less efficacy than what would be expected with Exzolt. The overall pharmacokinetics of Exzolt, which is formulated as an additive to drinking water, predicted that blood titers of fluralaner should be well above the concentrations necessary to kill 100% of the bed bugs throughout 21 days of the experiment, the end point of the Exzolt pharmacokinetic study [12]. This motivated us to conduct a pharmacokinetic analysis of Bravecto-treated hens.

Overall, bed bug mortality tracked the fluralaner concentrations in chicken blood. When concentrations were well above the LC_90_ value (days 2, 7.5, 9 and 14) nearly 100% of the bed bugs died. Conversely, when plasma levels of fluralaner approached the LC_50_ threshold (days 0.5 and 28) bed bug mortality dipped to the lowest levels (but still > 50%), with substantially greater variation among the replicates. The most revealing parameter was the peak concentration (*C*_max_), which was 106.4 ng/ml at 9 days post treatment (*T*_max_), compared to 355.1 ng/ml at 7.5 days with Exzolt [12]. Interestingly, the mean half-life (HL_λz) of Bravecto-formulated fluralaner in chickens was 7.1 days, rather similar to the 5 days reported for Exzolt-formulated fluralaner after intravenous administration [16]. These results suggest that the gavage treatment with Bravecto was less effective than with Exzolt at delivering fluralaner to blood. Thus, peak concentrations of fluralaner were damped, but its persistence in blood appears to be similar to that in the Exzolt treatment.

Interestingly, recent evidence suggests that upon strong selection with fluralaner, filth flies rapidly evolve high levels of resistance to fluralaner [17, 18]. Therefore, its use in poultry farms should be judicious, and systemic approaches (xenointoxication) for managing hematophagous pests should include rotation of ectoparasitic drugs with different modes of action. Nevertheless, even at > threefold lower titer than expected, fluralaner was highly efficacious against bed bugs, and as a systemic treatment it represents an innovative and possibly transformative technology to eradicate bed bug infestations from poultry farms.

The similar mortality results we saw across our two independent studies and the strong association of mortality with plasma concentration was in contrast to a recent study that used the same treatment regime of chickens (Bravecto orally administered to chickens twice at 0.5 mg/kg body mass each) and examined the mortality of kissing bugs that fed on the medicated chickens [19]. Surprisingly, in that study fluralaner concentration was highly variable across the three replicates and over time, reaching below the LOQ (2.5 ng/ml) by day 28 [19].

Bed bugs must feed to be able to molt to the next developmental stage and to produce eggs. Therefore, depending on the temperature and the size of their previous blood meal, bed bugs generally seek a blood host and feed every 5–7 days. The results of bed bug mortality and fluralaner plasma concentrations indicate that the dual administration of fluralaner to chickens results in plasma concentrations well above those needed to kill bed bugs and for a time period that would cover multiple feeding events in the bed bug life cycle. Moreover, since the egg incubates for about 10 days, the first instar nymphs that emerge would be exposed to fluralaner upon their first feeding on medicated chickens, resulting in significant disruption of bed bug population dynamics.

## Conclusions

This study confirmed that fluralaner is highly efficacious as a systemic ectoparasitic drug against bed bugs in poultry. Based on the pharmacokinetics of fluralaner formulated as tablets (Bravecto) and administered by oral gavage, we expect that the administration of two doses of fluralaner via drinking water (Exzolt) at 0.5 mg/kg chicken body mass 7 days apart would be more effective against the common bed bug for more than a month. Innovative strategies to manage and eradicate bed bugs from poultry farms are sorely needed, and fluralaner could be a transformative component of IPM interventions in poultry production farms.

## Data Availability

The data sets supporting the results are available from CS (coby@ncsu.edu) upon reasonable request.

## Abbreviations

AUC_inf_: Area under the curve from time zero to infinity
AUC_last_: Area under the curve from time zero to the last time point
Cl_F: Clearance per fraction absorbed
CI: Confidence interval
DDT: Dichlorodiphenyltrichloroethane
DMSO: Dimethyl sulfoxide
λz: Elimination rate constant
AUC_extrap_: Extrapolation of AUC
IPM: Integrated pest management
LC_50_: Lethal concentration that killed 50% of the population
LC_90_: Lethal concentration that killed 90% of the population
LOQ: Limit of quantification
*C*_max_: Maximum plasma concentration
MRT: Mean residence time
*C*_max_: Peak concentration
RR: Resistance ratio
HL_λz: Terminal half-life
*T*_max_: Time to peak concentration
Vz_F: Volume of distribution per fraction absorbed
